# Men’s perceptions of sexual and reproductive health education within the context of pregnancy and HIV in Zambia: a descriptive qualitative analysis

**DOI:** 10.1186/s12889-021-11430-3

**Published:** 2021-07-08

**Authors:** Tulani Francis L. Matenga, Joseph Mumba Zulu, Sharon Nkwemu, Perfect Shankalala, Karen Hampanda

**Affiliations:** 1 Department of Health Promotion and Education, School of Public Health, University of Zambia, Lusaka, Zambia; 2 Division of Academic Specialists in Obstetrics and Gynecology, University of Colorado, Denver, USA

**Keywords:** Sexual and reproductive health, Antenatal care, Male involvement, Health education, Zambia

## Abstract

**Background:**

Although health care providers are beginning to focus on men’s roles as fathers and husbands, there is limited understanding of how men view their ability to promote sexual and reproductive health in families affected by HIV and their experiences with receiving education through antenatal care. This paper aims to explore men’s perceptions of the education they need regarding sexual and reproductive health within the family in the context of HIV.

**Methods:**

We interviewed a convenience sample of 18 male partners of pregnant women living with HIV in Lusaka, Zambia. Atlas.ti was used to facilitate data management and content analysis.

**Results:**

Men reported being the primary decision-makers regarding sexual and reproductive issues in the family; however, they admitted far-reaching unmet needs in terms of information on sexual and reproductive health in the context of HIV. Most men felt that antenatal care was not a conducive setting to fully educate men on sexual and reproductive health because it is a woman’s space where their health concerns were generally neglected. There was a strong desire for more education that was specific to men’s sexual and reproductive health, especially because all the couples were affected by HIV. Men especially requested education on sexual preparedness, safe sex, the use of condoms in sero-concordant and sero-discordant relationships and general health information. Although men stated they were the main decision-makers regarding sexual and reproductive issues such as pregnancy, most men were not confident in their ability to promote sexual and reproductive health in the family because of limited knowledge in this area.

**Conclusion:**

There is need to change the environment and messaging of antenatal care, as well as offer relevant education opportunities outside health facility settings to empower men with essential information for meaningful involvement in sexual and reproductive health in the context of HIV.

**Supplementary Information:**

The online version contains supplementary material available at 10.1186/s12889-021-11430-3.

## Background

Male involvement is a key strategy to promote sexual and reproductive health in the context of HIV [1, 2]. Male attendance at antenatal care (ANC) and participation in prevention of mother to child transmission (PMTCT) is associated with improved maternal health behaviours [3] and can reduce the risk of vertical HIV transmission and infant mortality by more than 40% [4]. Mweemba et al., [5] further argue that men as sexual partners contribute to the epidemiology of HIV through their engagement in risky behavior such as multiple sexual partners and through sexual violence. In this regard, concerted efforts have gone into developing strategies to expand PMTCT services that include male partners, such as offering couple HIV testing during ANC [6]. Despite these efforts, there are considerable barriers to meeting men’s sexual and reproductive health needs, including their lack of information and limited understanding [7, 8].

The lack of outreach and information to men represents an important missed opportunity for male partners to be involved in the promotion of sexual and reproductive health in families affected by HIV. Historically, men in low and middle- income countries (LMICs) have received very little attention in terms of promoting sexual and reproductive health, either from the research community or from public sector health care planners and providers [9]. This situation is predicated on the fact that women bear a greater burden of reproductive mortality and morbidity as they shoulder the responsibility for childbearing [10, 11]. Largely missing is information that could assist men in making decisions regarding the roles they could play to promote sexual and reproductive health within the family [12, 13]. Men need self-esteem, self-awareness and skills to promote good family health, to engage sexually in ways that are respectful of themselves and their partners; and to be confident to make sexual and reproductive health decisions for the family [14]. Ramirez-Ferrero and Lusti-Narasimhan [15] argue that to maximise the health outcomes of PMTCT and sexual and reproductive health programs for women and men, there is a need to move beyond seeing men as simply facilitating factors that enable women to access health-care services. Men need to instead be recognised as an essential part of reproductive health policy and practice.

It is not possible to achieve the global sexual and reproductive health goals without considering men’s knowledge and participation [16] as they are typically the primary decision-makers in many settings and directly affect their partner’s and children’s health [17, 18]. However, relatively little attention has been given to men’s reproductive and sexual health concerns [14]. In Zambia, a study conducted by Muloongo and collogues [19] to explore the perspectives of male participation in ANC in a military setting revealed that men were motivated to attend ANC because of the desire to get information about pregnancy and care; the desire to have a healthy mother and baby; the privileges couples receive (e.g., jumping the queue and being seen first); and the desire to be part of pregnancy decision-making. Most available literature on men’s reproductive and sexual health and their related needs have focused on men’s role in family planning and male involvement in ANC as a vehicle to prevent mother to child transmission of HIV [8], while neglecting other aspects of sexual and reproductive health. Involving men in decision-making regarding sexual and reproductive health requires they have appropriate knowledge and understanding of key sexual and reproductive health issues, including HIV [11]. This paper, therefore, focuses on men’s perceptions of the sexual and reproductive health education they have received, unmet needs, and the role of ANC in pregnancies affected by HIV in Zambia.

## Methods

Data reported in this paper come from a larger qualitative parent study aimed at refining a couples counselling intervention for pregnant women living with HIV and their male partners in Lusaka, Zambia [20] conducted between January – May 2019. The study recruited and interviewed a clinic-based sample of 30 pregnant women and 18 of their male partners, as well as conducted focus group discussions with health care providers. Here, we present and discuss themes related to the promotion of sexual and reproductive health from the interviews conducted with 18 male partners of the pregnant women living with HIV, similar works were also presented at the 14th Interest-2020 virtual conference [21].

### Participant recruitment

We recruited male participants through their female partners who were attending ANC at a large district hospital in Lusaka, Zambia. The targeted sample size for male interviews was 15–20 participants. The research team continued recruitment of female partners and male partners until the study reached theoretical saturation, which occurred with 30 women and 18 of their male partners. Of the 30 female partners who were interviewed, 12 women refused to provide consent to contact their male partners. Our research team worked in collaboration with nursing staff to invite eligible female participants living with HIV to learn about the study and potentially participate in an interview after accessing their ANC services. For those who came as a couple (7), after completing their ANC services, both the woman and her male partner were invited to participate in an interview in separate private rooms simultaneously by gender-matched interviewers. If the male partner was not present during the ANC visit (11), permission was sought from female participants to contact their male partner for a potential interview. If they consented, they provided their male partner’s phone number. The research team then followed-up by calling the male partner and inviting him for an interview on family health using a standardised recruitment script. The study was explained to potential participants as wanting to discuss issues related to maternal and child health, including HIV; participants were not made aware of HIV status inclusion criteria nor their partner’s HIV status at any point during the recruitment, consent, or interview procedures. Participants were offered the choice of an interview in a private room at the clinic or at another private location of the respondent’s choosing.

### Data collection

A total of 18 male partners completed an interview. Each interview was carried out in either English, Bemba or Nyanja by a gender-matched experienced qualitative Zambian researcher, with a background in public health/health promotion and education and with previous experience in qualitative and HIV research. Data was collected about the respondents’ sociodemographic characteristics and family health, couple’s relationship dynamics and perceptions about couple-counselling visits using a semi-structured question guide developed for the parent study [20] using existing literature (see Additional file 1). Interviews lasted between 20 and 60 min long. In this paper, we particularly focus on men’s experiences with and perceptions of the education they need regarding sexual and reproductive health within the family in the context of HIV.

### Data analysis

The interviews were audio-recorded, translated verbatim, and transcribed. We used a combined inductive and deductive coding approach that captured specific themes while leaving flexibility for new themes to emerge particularly in relation to majority perspectives, and previous experiences of participants [22]. All transcripts were discussed among the entire research team after finishing an interview to identify probes or follow-up questions to be included in the next interview. These reviews also helped to decide when theoretical saturation had been reached. To ensure data validity, the primary investigator (KH) verified and reviewed all data transcripts before the coding process. A codebook was developed after the interviews were completed in an iterative process through consensus and agreement of the research team by reviewing and discussing the transcripts and codes (see Additional file 2). The first author conducted the initial process of familiarisation with the data through review, initial coding and identification of major themes. The first, second and last author independently coded the first three transcripts and then met to refine codes and themes. The research team members collaborated through regular data meetings to finalise the thematic analysis of coded data. To facilitate data management and analysis, Atlas.ti was used, a qualitative software used to code (i.e. label), categorize, classify, store and manage the data for this project. Final themes related to men’s sexual and reproductive health education in the context of HIV included: benefits of ANC attendance, confusion and frustration with information, ANC as a woman’s space, ANC and men’s work schedules, men’s desired education to be able to promote sexual and reproductive health in the family.

### Ethical considerations

All participants provided written (or thumbprint) informed consent. The study procedures were approved by the University of Colorado Multiple Institutional Review Board (COMIRB), the University of Zambia Biomedical Research Ethics Committee (UNZABREC), and the Zambia National Health Research Authority (NHRA).

## Results

### Demographic characteristics of study participants

Table 1 displays the sociodemographic characteristics of the men we interviewed (*n* = 18), who were all partnered with a pregnant woman living with HIV. The majority, 11 of the male partners were also living with HIV (i.e., sero-concordant), while 7 reported being HIV-negative (i.e., sero-disconcordant). The average age of the participants was 40 years (range: 29–61 years). With 5 of the respondents having completed secondary school education.

**Table 1:** Characteristics of study participants

	Male (*n* = 18)
Age: mean (sd)	8.9
Married (yes): (n)	18
Length of relationship	
2 years or less: (n)	4
3–6 years: (n)	8
> 6 years: (n)	6
Parity: mean (sd)	–
Currently pregnant (yes): (n)	–
Number of children: mean (sd)	1.5
Highest level of edu.	
< Primary	1
Primary: (n)	9
Secondary: (n)	3
> Secondary: (n)	5
Can you read a newspaper?	
Easily: (n)	15
With difficulty: (n)	2
Not at all: (n)	1
Electricity in the home (yes): (n)	18
Cooking source mainly firewood (yes): (n)	2
HIV-positive status (yes): (n)	11
Disclosed status (yes): (n)	18

### Benefits of ANC attendance

All male partners recognised and endorsed the importance of accompanying their pregnant wives for ANC. Although not all male partner attended ANC with their female partners, those who attended ANC found that the experience provided them reproductive health education and the opportunity to learn more about how to look after their partners during pregnancy and how to prepare for the coming baby. There were more educated men who attended services with their partners, these men found the information useful. As the following quote reveals, men were also generally supportive of their partners’ participation in PMTCT services during ANC and felt it was their responsibility to be there during these visits and to provide support through HIV counselling and testing.

*It is very good to escort our wives when they are pregnant. When I escort her, I know her status, not just her coming to tell me at home. Therefore, the way I have come today, I had to do an HIV test because she is HIV positive … … . It better I am with her often so that I am there to encourage her to say, “Let’s go [for antenatal] and see what comes next”.* **HIV negative** – **35-44 years old- Secondary school level**

Several men, particularly young men, who were first time fathers also revealed that health talks during ANC can be important for male partners to learn how to ensure that the female partner remains healthy, has a safe delivery, safe baby feeding practices, and how to look after the baby. The following quote highlights one man’s views of the helpfulness of such information:

*There are certain things which I get from those talks like the things which men are supposed to do for a pregnant woman, such things I understand them, and I even have answers sometimes on how a pregnant woman is supposed to be taken care of*. **HIV positive** -**25-34 years old-Primary school level**

In addition, participants indicated that they received and appreciated the information on child spacing and family planning. Information on topics such as child spacing, and family planning was much appreciated by older men who had been married to their partners long and had more children. The following quote explains the information that one man received and how it was helpful to enable the infant to grow healthy:

*“ … they tell me about how to live with my wife at home [as a sero-discordant couple], that in order to have a healthy baby, my wife has to eat this type of food and you are supposed to do this but it’s not supposed to be all the time [sexual intercourse]. So, you are supposed to give her some time [between pregnancies] so that you take good care of the baby*”. **HIV negative** – **35**-**44 years old-Secondary School level**

### Confusion and frustration with information

However, many of the male participants who had attended ANC felt that most information during ANC was directed at pregnant women and excluded men’s role in sexual and reproductive health. Men generally felt that attending ANC did not adequately prepare them to help promote optimal pregnancy outcomes, including PMTCT. During our interviews, men discussed numerous misconceptions and incorrect information, which they reported receiving from health care providers. Confusion around promoting sexual and reproductive health in sero-discordant couples was especially salient. Many participants did not understand how one partner could be positive while the other was negative nor how to best prevent HIV transmission in such relationships, as the following quote highlights:

*I never got it [HIV], probably it was hiding, and probably it was in my blood. Probably it was these people, they used to take ARVs which made the thing weak so that I could not get it according to the information that you [health providers] give out. I also didn’t know how one partner could be positive and the other negative; most people should start knowing now*. **HIV positive**- **55-64 years old-Secondary School level**

Men explained that they were taught during ANC that couples should practice safe sex (i.e., use condoms) during pregnancy as a way to ensure that the unborn child does not get infected with HIV, which is not an effective PMTCT intervention among women who are already living with HIV. However, they were also taught about the (accurate) importance of condoms in sero-discordant relationships to prevent HIV-negative men from acquiring HIV from positive partners. Participants further reported that health education at ANC placed a large emphasis on using condoms to prevent HIV, but that there was a low level of buy-in from men. Men discussed doubts about the need to use condoms in relationships where both partners were living with HIV. Participants admitted that they did not understand why they should use condoms and did not view condoms as a feasible option in their relationships. They discussed that condoms are not typically acceptable in a marriage and are associated with having multiple outside partners and a sign of infidelity. Men also expressed confusion around condoms and childbearing in couples affected by HIV and did not understand how they can be expected to use condoms when they want more children. One participant explained that his questions regarding HIV and family planning were not well answered, leaving much confusion and frustration as to the promotion of sexual reproductive health:

*Sometimes when you go to the clinic, they give you condoms to use with your wife, but that sometimes becomes a problem, why would I use a condom when I want to have a child*?... *And that’s where I want to understand very well if I start using condoms, how I am going to live [have sexual relations] with my wife?* **HIV positive** *-***35-44 years old-University level**

The use of condoms was more confusing among HIV positive men as compared to HIV negative men as positive men did not see the need to use them because their female partners were living with HIV. This is evidenced in the above quota; it was more likely that HIV positive men had more questions regarding their health as compared to their counterparts who were HIV negative.

### ANC as a woman’s space

Among the men who attended ANC, many indicated that during ANC health talks, only a few men were present, which made them feel uncomfortable and out of place. This feeling of being uncomfortable was described as a major challenge to men’s active participation in the health talks, where men felt they could not freely ask questions because it was a women’s space. This is highlighted in the quote below with a male partner who was uncomfortable being in a space with 50 women and only a few men:

*I came on Monday; I escorted my wife. If the floor is open, where they are educating them on how to keep the pregnancy, how to prepare for the incoming children or child. You see, those are open they say, “please you come with your husbands”. And the health care providers were very happy because we were just about 4, 4 men who escorted their wives out of more than 50, that room was fully packed but out of that we were only 4 men. The curtesy they gave to us was very nice. Even though some I could read for myself, from experience, I could read that that one is feeling shy, the other one is not comfortable, he is just seated in the corner, he can't even look around, he can't even ask. The nurse says any question men's time for questioning, nobody, no everything is ok (small laugh) which was very bad. So, we are lacking knowledge.* ***HIV positive*** *–* ***55-64 years old-Secondary School level***

For those men with secondary school level education, ANC attendance was seen to be important, despite them being present, they still felt uncomfortable and not belong there because of a large presence of women.

### ANC attendance and work schedules

During our interviews, men who were educated up to university level and had formal jobs, explained that they are the bread winners in the family and need to engage in income-generating activities. Even if they wanted to accompany their female partners to ANC, health services are often only offered during working hours. Men who did not accompany their wives for ANC services interviewed at home or other locations raised this concern, which they perceived to hinder them from playing a significant role in their family’s sexual and reproductive health. The quote below with one participant who emphasised the important role employers could play in giving permission to male partners to support their wives during pregnancy expresses this challenge:

P: *It should be from the employer’s side; men should be given some permission. So, it follows the workplace, if they are not given permission, they can’t come.*

I: *So, you are saying the workplace is a challenge?*

P: *Yes, that’s a challenge because if he misses work, his pay will be cut. His employers will not understand that he went for antenatal, they would ask him “are you the one who is pregnant?”* ***HIV positive*** *-* ***35-44 years old- University level***

### Fear of HIV testing

An additional barrier to male attendance at ANC reported during our interviews was the fear of HIV testing and disclosure within couples. Male participants reported that men are hesitant to come to the clinic because they believe they will be forced to test for HIV, as the following quote explains:

*For a man to declare himself that “no they want me at the hospital?” And he says “yes” ahh “what for?” He doesn’t want, just for a few questions that they will [ask], he’s fearing to be asked are you [HIV] positive or not, or to be tested if he’s not tested, they have to test the woman and they have to test the husband. So those things, that’s why am saying we are lacking knowledge. That knowledge we don’t have that. So, we need a lot of sensitisation*. **HIV positive** – **55**-**64 years old-Secondary school level**

This fear of testing at the health facility could also explain the few numbers of men escorting their pregnant wives for ANC as most men associate health facilities with HIV testing with many fearing HIV positive results.

### Desired education

Overall, our participants explained that they felt as though they did not have enough information about sexual and reproductive health in the context of HIV, and as a result, reported low self-efficacy to promote their family’s health. Men also emphasised that a space for male partners to have their questions answered during a pregnancy – especially a pregnancy affected by HIV – is critically important, as the below quote highlights:

*I have more questions than the answers which I have. So, if I could go deeper into conversation like this one, I think that would be better for me and my family*. **HIV positive** – **55-64 years old- Secondary school level**

Participants also indicated that men needed more education on sexual behaviour and HIV to prevent infection and reinfection to other sexual partners:

*Men need more sensitisation and to educate them on how to prevent HIV because some, they just feel when someone is positive you just start sleeping around with each and everyone not knowing that you are just re-infecting yourself. So the more you go out sleeping around like that the more you are re-infecting yourself so there is much more work needed to sensitise about that because some men they don’t have that knowledge, they think that when you are positive then you will die within days, you just have to take care of yourself properly, eat good food, drink a lot of water and take rest specifically*. **HIV positive** – **25-34 years old- University level**

Male participants felt that they needed more information, especially in discordant couples, on PMTCT. One participant described how he did not feel confident in preventing infection to the unborn child given that he is HIV-negative, and the wife is HIV-positive:

*What I can say about our family health from my point of view is that, since my wife is HIV positive and am not. So, I don’t know how we can keep our baby so that we can protect the pregnancy so that she can come and deliver safely. So, this is what I would like to know*. **HIV negative** – **35**-**44 years- Secondary school level**

Some male participants further highlighted the need for sexual reproductive health education to extended beyond keeping a pregnancy healthy and emphasise respect for female partners:

*Those are the areas which should be touched not after [sexual intercourse], you just wanted to sleep with that person and dump. That is what men should be educated about. Some like other components, our partners they are not, I mean they are not just there like sexual objects. They are human beings like us. So, men should be educated on that one. They don’t look like they are just objects to use and dump*. **HIV positive**- **35**-**44 years- University level**

Young men also revealed that it was important for men to have more information about HIV and PMTCT beyond what is provided by health care providers during the ANC health talks, including relationships that are both sero-concordant HIV-positive and sero-discordant:

*It's very important to teach people about such sexual and reproductive health because like for me I had no idea that something like that would happen [being HIV positive]. So, like what they tell us that we have to prevent our women from diseases like HIV when she's pregnant, we have to prevent the baby in the womb not to contract the virus which we have*. **HIV positive** - **25-34 years old-Primary School level**

## Discussion

This study revealed men’s perceptions and needs regarding sexual and reproductive health education in Zambia and the role of ANC in adequately providing information. The results show that while all men appreciate the importance of accompanying their female partners for ANC, men experienced several barriers to attendance, including the hours during which these services are offered and fear of testing and disclosure of HIV status. Generally, men displayed low levels of confidence in relation to their ability to promote HIV-related sexual and reproductive health within the family. In this regard, men desired more education on sexual behaviour and HIV to prevent infection and reinfection to their sexual partners and how sero-discordant couples can continue having healthy sexual relations and more children. Men also expressed a desire to have more education on PMTCT in both sero-concordant and sero-discordant relationships.

Our findings show a lack of health information and education that most men need to effectively promote sexual and reproductive health within the family, which could help them to engage sexually in ways that are respectful of themselves and their partners. Contrary to findings by Kura and colleges [23] who reported that the majority of men viewed ANC as an important service but did not fully support their wives in utilising these services, men in our study generally understood and endorsed the importance of accompanying their pregnant female partners for ANC and the need to be aware of PMTCT, this finding is similar to that of Nkuoh and colleagues [24]. Our category of men ranges from 25 to 34, 35–44 and 45+ years, young men in our study, who were first time fathers found it necessary to attend ANC and get more information that would help them ensure a healthy pregnancy for their wives. The fact that these young men where first-time fathers explains why they appreciated health education provided during ANC than older men. Several men reported positive benefits, such as health information about safe delivery and items needed for delivery, safe baby feeding practices, and how to look after the baby. Similarly in a study by Davis et al., [25] male participants were motivated to attend ANC by a sense of shared responsibility for the baby’s health, by a belief that attending the clinic together will be good for the baby’s health, or by love for their pregnant partner.

Despite the appreciation for the information provided during ANC, our study found that men reported many unanswered questions and concerns regarding the promotion of sexual and reproductive health within their families. For instance, use of condoms was more confusing among HIV positive men as compared to HIV negative men. HIV positive men did not see the need to use condoms because their female partners were living with HIV. HIV positive men in our study were found to have less health information on HIV transmission dynamics and PMTCT. Clearly accurate, accessible information to make informed choices and safe, pleasurable sexual relationships possible would be best delivered through education and trained health professionals [26]. Men also explained that they were taught during ANC that couples should practice safe sex (i.e., use condoms) during pregnancy as a way to ensure that the unborn child does not get infected, which for women already living with HIV, is not an effective PMTCT measure. This was unsatisfying for the men we interviewed because they felt that condom use is associated with casual partners, infidelity and distrust, which led to poor uptake in married couples. This finding has similarly been reported in many contexts in sub-Saharan African countries [27, 28].

In our interviews, men described numerous challenges to receiving appropriate education on sexual and reproductive health through ANC. During ANC health talks, only a few men were present for these talks, which made those who were present feel uncomfortable and out of place. The knowledge that few men attend ANC is a barrier to men wanting to come because they fear feeling uncomfortable. Similar findings are reported by Leichliter and colleges [29] and Fleming and colleges [30], who reveal that men perceive health facilities as spaces for women where they are likely to experience disrespect and lack of confidentiality, this could explain why some men fear ANC services, such as HIV testing. Contrary to findings by Makoni et al. [31] in Zimbabwe that the friendliness and welcoming of male partners by health workers improves male involvement in PMTCT, men that attended ANC in this study reported feeling generally disempowered to ask specific questions regarding sexual and reproductive health because it was such a female-dominated space – also contributing their unmet need for information. This feeling of being uncomfortable as a barrier to men participating in ANC was also reported by Davis et al., [25] where prevailing gender norms and dominant forms of masculinity may contribute to men feeling too shy or ashamed to publicly show support for their pregnant partner.

In settings such as Zambia, cultural dynamics make it extremely difficult for men to feel comfortable asking questions about sexual relations in mixed-gender settings, especially in such an overtly female-dominated space like ANC. Thus, the historic institutionalisation of reproductive health, and particularly maternal health, as a women-only realm has yielded health services that continue to not accommodate men, contributing to men’s perception of clinic spaces as ‘women’s spaces’ and reproductive health as ‘women’s health’ [15, 24, 32, 33]. This study concurs that the traditional emphasis on maternal health has resulted in men being neglected in much of the sexual and reproductive health education [34]. Another challenge to male participation in ANC services reported in our interviews is that the services are conducted during working hours when men are supposed to be generating an income for the family, as others have observed [31, 35]. This challenge is directly conflicting with employers who may not want their employees to miss work. When ANC services’ operational hours are in conflict with the time during which men generate their income to support their families, it becomes difficult for men to participate in such services. This finding is similar to other studies which show perceived low accessibility to attend ANC services among men [24, 36, 37]. Men indicated that they wanted more information to be provided to them on issues related to their sex life because of being HIV-positive or having an HIV-positive sexual partner, and how they could take the necessary precautions to ensure that their baby is born healthy and HIV negative. Men who were not living with HIV also greatly desired more education on how to remain HIV-negative. Even though information on the prevention of mother to child transmission is provided to couples during health talks at ANC, male participants in this study felt that it was inadequate and that they needed more information, especially regarding sero-discordant couples.

The lack of men’s sexual and reproductive health information makes them have low confidence regarding their sex life especially when they are a discordant couple. This is evident from our participants’ mixed views on condom use, and how some felt they could not continue having sexual relations with their partners if they must use condoms, with others viewing it as a preventive measure and some not being sure it was the right thing for them. Health promotion outside of ANC is therefore needed to empower men with essential information for meaningful involvement [34]. Our findings support the conclusions of Kura et al. [23], who report that inadequate knowledge, cultural factors and lack of appropriate services adversely affect men’s involvement in their wives and their own sexual and reproductive health. In our interviews, men indicated they often felt sidelined by maternal health services provided during ANC clinics coupled with limited health information and knowledge to help them make informed choices and decision to protect and promote their own health as well as that of their families. This findings explains why there has been little evidence of any impact on antenatal support as others have observed [38].

This study suggests that education outside of the current ANC health talk model is needed for male partners to freely discuss their different situations and how to go about enjoying a healthy sexual relationship with their HIV positive partners without fearing being infected or infecting the unborn child. Based on these findings, we recommend incorporating health talks specifically for men into health care practice guidelines, educating them and providing gender-specific services, and providing condoms along with appropriate educational materials. In the context of care, services might be delivered in ANC settings or through community-based programs for those unable to attend as others have reported [31]. Men can be involved in the promotion of sexual and reproductive health as supporters of their partners, encouraging and enabling women’s utilisation of and access to services; as clients of health services, to help ensure their reproductive health and that of their partners; and as change agents in their communities [15].

We found the challenges that men face in fully participating in antenatal activities is because of the configuration of health care services. Educated men were more likely aware of the importance of ANC attendance and the usefulness of the education and information received. Men with university level education had formal jobs found it very difficult to make time to attend ANC as they had to be at work during working days. To overcome such challenges, we propose the integration of men’s sexual reproductive health in ANC services, which should include education and communication programs targeting men as well as tailoring the PMTCT programs and decentralising ANC services. When men acquire new knowledge and skills, they become actively involved in learning about and addressing health issues [39]. Unfortunately, public health institutions and the cultures in which they operate have unwittingly sustained a paradigm that associates sexual and reproductive health with women, and as such, have inadvertently excluded men [15]. As a result, men feel ill-prepared to promote sexual and reproductive health in their families and struggle to understand how they can have a healthy family in the context of HIV.

Studies further indicate that men’s sexual and reproductive health needs are not homogenous across social, cultural, economic and demographic characteristics [40] as they are characterized by age, ethnicity, sexuality, income, educational status, occupation, geographical location, position within a family, access to information and ability to put such information to use [9]. For example, younger men were more interested in information regarding how they could keep their wives healthy and learn safe baby feeding practices. On the other hand, information on topics such as child spacing, and family planning was much appreciated by older men who had been married to their partners longer and had more children. This indicates a clear need to consider the social demographic factors of men regarding sexual and reproductive health needs.

### Study limitations

This study provided important information on men’s reproductive health needs in the context of HIV. However, we cannot generalize these findings beyond the study setting as it was conducted with a small sample of respondents in an urban setting in the capital city of Zambia. Further research is therefore needed to find out if men in other settings report similar themes regarding sexual and reproductive health information and needs. A further limitation of the study is the generalizability to other populations of men, which is a limitation of most qualitative work given the small sample sizes and often highly specific social characteristics. Our study is also potentially affected by social desirability bias where respondents were inclined to give more favourable answers.

Our study also recognizes that men are often reluctant to participate in research and particularly difficult to recruit especially in places considered as women’s spaces (ANC/health facilities) because of their busy schedules related to work or income generating activities that they are involved in. In our study, men were at times in a hurry at the health facility and recruiting them through their female partners in some cases proved to be a burden for women as they had to convince them to have the interview. This in itself may reinforce notions that women are responsible for reproductive health by being the point of access for recruitment. To overcome this challenge, we offered men an option to have the interview in a place of their choice such as the home over the weekend or any other place they felt comfortable to have the interview. The interviewer at times had to be selective of the questions to be asked due to the limited time men had to give the interview. Despite the time constraint, most men enjoyed having someone attentively listen to their views, and the interview provided opportunities for them to express their knowledge on reproductive health issues. We therefore call for more research with male partners on reproductive health issues as Law [41] also observes.

## Conclusion

In conclusion, men in this study reported a lack of necessary knowledge and education as decision makers in the household to promote sexual reproduction health for them and their families, including PMTCT. Men desired more education on sexual and reproductive health in the context of HIV but felt that ANC may not be the best platform to delivery this information. The ANC environment remains an intimidating place for male partners both in terms of the physical space and messaging. Unique approaches to appropriately educate and engage men are urgently needed if the fight towards HIV is to be won, including eliminating mother-to-child transmission of HIV in sub-Saharan Africa by 2030 and meeting the ambitious UNAIDS’ [42] 90–90-90 target. There is an ongoing need for programs in setting like Zambia to address the specific health needs and concerns of men, as well and that focus on improving service delivery to accommodate men in the advancement of sexual and reproductive health in the context of HIV.

## Supplementary Information

**Additional file 1.** In-depth interview guide – male partner, Interview topics for male partners.

**Additional file 2.** Codebook for K99 qualitative data. Codebook developed for the parent study.

## Data Availability

The datasets used during the current study are available from the corresponding author on reasonable request at matengatulani@yahoo.com

## Abbreviations

ANC: Antenatal Care
PMTCT: Prevention of Mother to Child Transmission
