# Supplementary material for: Men’s perceptions of sexual and reproductive health education within the context of pregnancy and HIV in Zambia: a descriptive qualitative analysis
Source: BMC Public Health. 2021 Jul 8;21:1354. doi: 10.1186/s12889-021-11430-3 (PMC8268604; doi:10.1186/s12889-021-11430-3)
Supplement: Supplementary file 1 — Additional file 1. In-depth interview guide – male partner, Interview topics for male partners. [file 12889_2021_11430_MOESM1_ESM.docx]

## In-depth interview guide – male partner

#### I. INTRODUCTION

My name is ___________________. I am working with the University of Zambia School of Medicine and the University of Colorado, Denver, in the United States on a project aiming to improve communication and health behaviors in Zambian couples. We would like to talk to you about your perceptions of couples communication and decision-making, family, health, and couples counseling. Everything you share during this interview will be kept confidential and will not be shared with your partner or anyone outside of the study team afterwards. The information that you provide will be used to inform efforts to strengthen and improve health services in Zambia.

Remember, you don’t have to talk about anything you don’t want to and you may end the interview at any time. This interview will take around one and a half hours. If you have questions you want to ask on other topics, I can assist you to find answers after the interview is over.

(***Go through the informed consent form for in-depth interview participants out loud*** and give the participant a copy. If she agrees to participate, ask her to sign the informed consent form. **Complete the participant characteristics form.** Ask permission to tape record the discussion, and if she agrees, start the tape recorder AFTER the completion of the participant characteristics form and the introductions part of the discussion. This guide includes the topics to be covered and questions that may be helpful in facilitating the interview. You do NOT have to ask all the questions or follow the order given in the guide.)

*II. DISCUSSION TOPICS*

1. **Family Health**
   1. What types of information about family health do you think is the most important during and after pregnancy?
      1. Do you feel like you got all the information you needed to help your wife/partner have a healthy pregnancy? Why/why not?
      2. Where did this information come from?
      3. What other information would you have liked to get?
      4. Who would you have liked to get this information from?
   2. Do you and your partner/wife talk about health issues and ways to make sure that the family stays healthy? What do you discuss?
   3. How do you show that you are concerned with the family’s health? Do you think most men do this?
   4. what ways can men be involved in family health during pregnancy?
   5. what ways can men be involved in family health postpartum?
   6. What things specifically do you discuss with your wife/partner that are related to health concerns in the family?
   7. What do you think is most important for your family’s health right now? Why is this the most important? Is there anything else health-related for you, the baby, or your partner/wife that you are concerned about?
   8. Tell me about how you and your partner discuss issues related to HIV?
      1. Have you ever been tested together? Do you know each other’s status?
   9. What do you think are the best approaches to discuss issues surrounding HIV as a couple?
      1. What things would help you be more confident discussing this topic with your partner/wife?
      2. What ways do you think we could encourage men to be involved in their family’s health, particularly around issues related to HIV?
      3. What do you think about meeting with a mentor/counselor to help give you advice on health issues either by yourself or with your female partner/wife?
      4. What ways do you think would help men be interested in such a program?
2. **Couple relationship dynamics**
3. Can you tell me how a wife and a husband show respect for one another?
   1. Can you give me an example?
4. How do you feel that your husband/partner shows you respect?
5. How do you feel that you show your husband/partner respect?
6. How do you talk with or communicate with your wife/partner?
   1. Who usually initiates the conversations?
   2. What do you talk about?
   3. How often do you take to each other?
   4. What are the main things you discuss? Why?
   5. How do you know that your partner listens to you or cares about what you are saying?
7. When you talk with your partner, do you ever hide information? What do you hide? Why?
8. Is there trust in your relationship with your wife/partner?
   1. Can you give some examples how you know you can trust your partner (or examples of why you do not trust her)?
9. Can you tell me why or why not you are satisfied with your relationship?
   1. What things does she do that make you happy?
   2. Does she do anything that makes you mad?
   3. Are there any things you wish she would change about her behavior?
10. In your relationship/marriage, how are important decisions usually made?
    1. Do you feel like you make decisions together or does one of you usually have the final say? How does this make you feel?
    2. Do you think most Zambian couples make decisions this way?
11. When there is a problem that you are facing, how do you work to solve that problem? Do you seek the help of your wife/partner?
    1. Do you work together to solves problems? How?
    2. Do you think most Zambian couples solve problems this way? Why/why not?
12. When you disagree with your wife/husband, how do you resolve conflicts?
    1. Can you give me an example of a conflict you had and how you and your partner/wife worked to resolve it?
13. Will you explain if you or your partner ever say cruel or hurtful things when you disagree or are having an argument? Why do you think this happens? What would help couples to not insult one another when they disagree?
14. Will you explain if you or your partner ever use violence (hitting, throwing things, etc) during a conflict? Why do you think this happens? What would help couples who use violence stop?
15. **Counseling visits**

*We* *would like to develop an intervention for pregnant women and their male partners to strengthen relationships and promote family health. In order to make sure that the intervention is successful, we are talking to men about our ideas for this program.*

1. What ways do you think we can get women and men interested in participating in a program about family health and communication within couples?
   - 1. What things would couples be interested in learning about?
2. What barriers might we encounter?
   - 1. How can we overcome those barriers?
3. Would you be willing to participate in a couples intervention, meeting with your wife/partner to learn about health topics and things to make your relationship stronger? Why/why not?
4. How frequently do you think couples would be willing to come together with a counselor/mentor/educator (every week, once a month, etc)?
5. What do you think would be the best location (the home, the clinic, an office space) for a couples intervention, where the coupe meets with a counselor/mentor/educator to discuss health topics and work on skills that will make the relationship stronger?
   - 1. What would be some of the advantages of meeting in the home?
     2. What would be some of the disadvantages of meeting in the home?
     3. What would be some of the advantages of meeting elsewhere, such as a designated office space or at UTH?
     4. What would be some of the disadvantages of meeting elsewhere, such as a designated office space or at UTH?
6. What types of health information do you think couples would be the most interested in learning during these meetings?
7. What types of information and skills regarding the relationship do you think should be provided?

PILE SORTING ACTIVITY:

We have some ideas about topics to discuss with couples during the couples counseling. Looking at the cards in front of you, Can you please put them in order from what you believe would be the most important to the least important:

- - 1. Communication skills?
    2. Respect?
    3. Honesty?
    4. Decision-making skills?
    5. Conflict resolution skills?
    6. Support

******INTERVIEWER BE SURE TO READ OUT LOUD THE ORDER CARDS ARE RANKED AND DISCUSS WITH PARTICIPANT WHY THEY RANKED THEM IN THIS ORDER******

1. What additional topics do you think should be included in the session?
2. What do we need to be careful about when designing this program?
3. How can we ensure that our intervention best meets individual couple’s needs? What other things should we look at when designing the program?
4. What issues should we make sure we address in order to help couples have stronger relationships?
5. What types of couples might not what to participate? Why?
6. What type of counselor/mentor/educator would you be the most comfortable with? Who do you think would be best to deliver information to the couple? What type of characteristics should these mentors/educators have? Would most people prefer to meet with a woman, a man, or both?
7. What do the mentors/educators need to be careful about when working in the community?
8. Do you have any other suggestions for a program that could help couples have stronger relationships and improve health?
9. Not all couples will receive the intervention we are developing. Some other couples may get education on another topic. Do you have any suggestions on what topics couples might like to learn about?
10. Are there any other things you would like to tell us about things or programs that you may have heard of or that you think can help families stay healthy and happy?

*IV. CLOSING*

Thank you very much for your time. Your responses will be very helpful for improving the health of Zambian families.
