# Supplementary material for: Men’s perceptions of sexual and reproductive health education within the context of pregnancy and HIV in Zambia: a descriptive qualitative analysis
Source: BMC Public Health. 2021 Jul 8;21:1354. doi: 10.1186/s12889-021-11430-3 (PMC8268604; doi:10.1186/s12889-021-11430-3)
Supplement: Supplementary file 2 — Additional file 2. Codebook for K99 qualitative data. Codebook developed for the parent study. [file 12889_2021_11430_MOESM2_ESM.docx]

**CODEBOOK FOR K99 QUALITAIVE DATA**

| **Major Themes (Section of Guide)** | **Sub-Themes**  **(Questions from Guide)** | **Themes from Interviews** | **Code definition** |
| --- | --- | --- | --- |
| Family Health | Perceived Importance of health Information | Nutritional information | Respondents’ perceptions about what kind of information is important to the health of the family and mother/baby, including that having to do with PMTCT |
|  |  | Preventing transmission to unborn child |  |
|  |  | Wellbeing of the child |  |
|  | Needed health information | Prevention of transmission to the unborn child | Apply text to which respondents talks about needed information during or related to antenatal/post-natal period |
|  | Role of Men in Family Health and PMTCT | Providing food | Apply to text about perceptions of what the male’s role is or should be in the health and well-being of their family as well as anything about what he does for the family in general |
|  |  | Helping with household chores |  |
|  |  | Encouraging partner to take medication |  |
|  |  | Showing compassion |  |
|  | Health related discussions | Use of condoms | Apply to text in which respondents talk about health-related discussions. Include text in which they do NOT discuss health related issues |
|  |  | Child spacing |  |
|  |  | Ensuring family stays healthy |  |
|  | Discussing issues related to HIV | Normal part for the couple/with easy due to acceptance | Apply text to which couples discuss health related topics and types of health related topics. Include text where they do NOT |
|  |  | Difficulty with HIV disclosure | General information about HIV disclosure experiences |
|  | Strategies to promote MPI in family health | Provide information during antenatal visits | Apply to text in which respondents share their opinions of strategies to promote male partner involvement in family health |
|  |  | Educate men |  |
|  |  | Engage employers |  |
|  |  | Providing counselling |  |
|  |  | Offering services over the weekend |  |
|  |  | Peer sensitization |  |
| Couple's relationship dynamics | Respect |  | Information shedding light on the couple’s relationship and general relationships functioning |
|  | Communicate |  |  |
|  | Trust |  |  |
|  | Decision making |  | Apply text to which respondents talk about how they make decisions in their relationship. Include text where one partner is responsible for making decisions |
|  | Conflict resolution |  |  |
|  | Support |  |  |
|  |  | Escorting partner for antenatal visits |  |
|  | Problem solving |  |  |
| Counselling visits | Types of information to be provided | Health information |  |
|  |  |  |  |
|  |  |  |  |
|  | Strategies to encourage participation in the intervention | Providing transport | Apply text to which respondents talk about the counselling visits/intervention without either same or both genders specified |
|  |  | Following couples home |  |
|  |  | Provide intervention during weekends |  |
|  | Barriers/Challenges | Clients not showing up |  |
|  |  | Level of willingness |  |
|  |  |  |  |
|  | Preference for the counsellor |  | Add text to which respondent describe preference for types of counsellors to deliver couple counselling |
|  | Relationship topics perceived to be most important |  | Add text to which respondents talk about topics perceived to be most important |
